# Inequalities of pandemic proportions

**DOI:** 10.1111/tct.13361

**Published:** 2021-05-07

**Authors:** Esther Dami Okhiria, Katherine Roxanne Rahnejat

**Affiliations:** ^1^ School of Medicine University of Sheffield Sheffield UK

In 2020, the world experienced two pandemics, namely Covid‐19 and racism. Admittedly, only one was ‘novel’ or ‘unprecedented’. On 23rd March, the British Government introduced a state of lockdown to reduce the spread of coronavirus.^1^ Subsequently, our medical school shifted education online by ceasing clinical placement and face‐to‐face teaching. As medical students, this left us uniquely affected; identifying as part of the National Health Service (NHS) yet asked to stay home.

In 2020, the world experienced two pandemics, namely Covid‐19 and racism.

We were keen to volunteer during the pandemic to reduce burden on the NHS, but as Black, Asian and minority ethnic (BAME) students we faced an additional obstacle—our heightened vulnerability to the virus. Over time, it had been shown that BAME patients suffered disproportionately higher mortality rates than their White counterparts.^2^ Covid‐19 was not only replicating existing health inequalities, but perpetuating them. Upon returning to clinical duties, we found limited welfare provisions (such as well‐being workshops or personal tutor support meetings) available to alleviate students’ ethnicity‐related health anxieties. As medical students, we feel a duty to help the sick, knowing we may become ill ourselves. Nonetheless, no one anticipated that ethnicity could influence our likelihood of dying from a novel virus.

Amid lockdown, the tragic death of George Floyd saturated the media with first‐hand stories of Black trauma.^2^ This incited deeper reflection of racial issues, with many advocating for change and pledging to self‐educate on ‘Black issues’.^3^ Social distancing and increased media usage precipitated greater time for public introspection, catalysing momentary support for Black Lives Matter (BLM).^2^ During this time we relived racial trauma, revisiting our own personal experiences through the words of others. Despite this, we were required to continue our studies. Although our medical school made general welfare provisions (such as a fortnightly tutorial) during the pandemic, we found limited additional support was available for Black students in the wake of the BLM movement.

During the height of public support for the BLM movement, students united in solidarity through protests. However, participation during a pandemic is contentious. The right to peacefully protest freedom from racial discrimination conflicted with the newly illegal nature of public gatherings.^1^ As BAME students we encountered an ethical dilemma; whether to stay home or participate in activities that might contribute to a second wave pandemic, compromise our ability to practice medicine or impact our own health. We protested through open letters to faculty, advocating curricular changes, such as, decolonisation of the medical curriculum and clearer frameworks for reporting racial harassment for students on campus and clinical placement.

George Floyd's death and Covid‐19 health inequalities had the cumulative effect of creating a racially charged lockdown environment for BAME students. We advocate that such students are optimally positioned to educate others based on their experiences and guide initiatives to improve student welfare. In light of this, we raise concerns that barriers to BAME students’ well‐being were heightened during lockdown, and left Black students particularly unsupported. We believe there are actions that British medical schools can implement to improve BAME students’ well‐being. Foremost, racially sensitive welfare support should be widely available and easily accessible at medical schools and clinical placement. Medical schools require appropriate staff training to facilitate race‐based dialogues, and systems in place to ensure training translates to meaningful changes in pastoral care. Universities must acknowledge the increased barriers and psychological burdens encountered by BAME students and make concerted efforts to combat impediments to discussing racial issues with faculty. Although the BAME workforce underpins the NHS, we find limited representation at our medical school. This appears particularly true for Black doctors and results in few relatable mentors for Black students. It is therefore imperative that tutors, irrespective of ethnicity, feel equipped to lead conversations surrounding race, thereby unburdening students from initiating the discussion. Secondly, enhancing diversity within medical school faculty and curriculum is paramount to achieving a positive cultural shift in medicine. We therefore propose increased efforts should be made to recruit ethnically diverse doctors into pastoral and mentorship roles. Likewise, greater BAME representation in ‘patients as educators’ programmes would better prepare students for the diverse patient population they will serve. Finally, we advise medical schools to adopt and advocate the BMA racial harassment charter,^4^ which sets standards to prevent and address racial discrimination.

Enhancing diversity within medical school faculty and curriculum is paramount to achieving a positive cultural shift.

Approaching its anniversary, ‘Black‐Out‐Tuesday’ resonates in our minds, as the world digitally protested racial injustice.^5^ Although many universities posted a black square in virtual solidarity, it is unclear how many will uphold their promises in 2021. Black ethnicities are often under‐represented within medical education; we believe this makes the voices of Black students distinctly important in issues relating to diversity, equity and inclusion at medical school. As BAME students, we advocate that the holistic initiatives delineated above, led by medical faculty, are fundamental to achieving an educational environment where all students can breathe and thrive.

‘Black‐Out‐Tuesday’ resonates in our minds.
